# Prevalence of supplement usage and related attitudes and reasons among fitness athletes in the gyms of Kashan and its relationship with feeding behavior: a cross-sectional study

**DOI:** 10.1186/s13102-024-00940-3

**Published:** 2024-07-08

**Authors:** Fatemeh Moradi, Akram Yazdani, Faezeh Nematolahi, Seyed Mohammad Hosseini-Roknabadi, Nasrin Sharifi

**Affiliations:** 1 Research Center for Biochemistry and Nutrition in Metabolic Diseases, Kashan University of Medical Sciences, Kashan, 87159-7347415973474 Iran; 2 Student Research Committee, Kashan University of Medical Sciences, Kashan, 87159-73474 Iran; 3 Department of Biostatistics and Epidemiology, Kashan University of Medical Sciences, Kashan, Iran

**Keywords:** Prevalence, Attitude, Supplements, Athletes, Feeding behavior

## Abstract

**Background:**

The overuse of supplements among athletes is a widespread issue affecting the health of both male and female athletes. However, research on supplements usage among female fitness athletes is limited, and there is little information on the feeding behavior of fitness athletes who use supplements. This study aimed to fill the gap in knowledge by examining the prevalence of supplement usage and its related attitudes and reasons among fitness athletes in the gyms of Kashan. It further aimed to investigate the correlation between supplements usage and the feeding behavior of fitness athletes.

**Methods:**

For these purposes, in this cross-sectional study, 433 fitness athletes (15‒46 years old) in 28 gyms in the city of Kashan were surveyed using a researcher-made questionnaire in 2023. Five experts confirmed the validity of the questionnaire. The present study considered the supplements based on the Australian Institute of Sport position statement. A Chi-square analysis was conducted to examine the relationship between the study variables and supplement usage.

**Results:**

Overall, 272 male and 161 female fitness athletes participated in this study. The results revealed that 57.9% of participants used supplements, most commonly vitamin C, vitamin D, omega-3 fatty acids, and whey protein. The main reason for using supplements was to speed up body repair after exercise (69.5%). Additionally, 41.8% of these athletes believed that using supplements improves their overall performance, and 21.9% thought that supplements do not harm the body. Moreover, a correlation was observed between feeding behavior and the consumption of supplements. It was found that athletes who use supplements tend to eat more white meat, seeds, and nuts and fewer high-fat dairy products than those who do not consume them.

**Conclusion:**

Using supplements among fitness athletes in the gyms of Kashan is common. The main reason for using these substances was to speed up body repair after exercise, and nearly half of the athletes believed that supplements improved their performance. In addition, it was revealed that athletes who take supplements have healthier feeding behaviors than those who do not. Thus, these findings confirm the necessity of informing fitness athletes about using supplements.

**Supplementary Information:**

The online version contains supplementary material available at 10.1186/s13102-024-00940-3.

## Background

Athletes have been utilizing various strategies to gain a competitive advantage since adolescence, due to the development of novel training methods and media depictions of professional sports. Sports nutrition is the synthesis and application of nutritional and exercise physiology principles based on science to improve physical activity, sports performance, and recovery. In addition to implementing training and sports nutrition strategies, athletes also seek out certain supplements [1, 2]. Considering that athletes have more significant nutritional needs than others, which increase with time and increase the intensity of athletic activity, more substances are required to provide the metabolic needs of athletes during training or the recovery period. Particularly for athletes with massive bodies, consuming the correct and sufficient amount of food to satisfy all their daily needs and calorie intake is challenging. Many athletes tend to use supplements [3]. Supplements are commercially available products that are intended to complement the diet to improve overall health, promote faster recovery, and boost athletic performance [1, 4]. It is notable that there is no single and universal definition of a supplement because of the lack of a unified classification system or a controlling approach to these products. According to the Australian Institute of Sport (AIS) statement, a supplement is a single or multi-ingredient product in powder, limited volume liquid, pill, or capsule form providing nutrients or other dietary components to achieve a specific health and/or performance benefit [5]. They include vitamins, minerals, herbs, amino acids, ergogenic aids, and various other products [6, 7]. At one end of the spectrum of supplements are typical foods, while at the other end are clearly identifiable drugs; however, between these two ends, there are several compounds that are difficult to classify. Nevertheless, supplementation is usually a term applied to specific nutrients or compounds rather than whole foods [8, 9].

The use of supplements has increased dramatically in the last decades. However, there is a limited body of scientific research on their safety, quality, and effectiveness [10, 11]. Today, excessive consumption of supplements is a prevalent social issue that can lead to health problems and poor performance for athletes [2, 10–14]. The efficacy of many supplements is controversial, whereas the side effects are clear [9]. Some supplements may have adverse health effects, such as a significant number of cardiovascular and central nervous system complications, liver disease, and pancreatitis, but the identification of side effects can be challenging [15–18]. Products are often removed from the market only after the occurrence of a significant number of damaging events [3, 16]. Studies have revealed that many athletes consume excessive amounts of supplements even when they do not require them [19]. More knowledge is required regarding supplements due to their increasing consumption [13]. Unfortunately, athletes rarely seek information from knowledgeable sources, such as dietitians. Furthermore, education programs on this subject are unavailable in every country, especially in developing countries. This leaves athletes vulnerable to misinformation, which can harm their health and lead to misunderstandings about their sport [14]. Unintentional use of supplements is also a risk factor for the consumption of illegal substances and may cause “involuntary doping” due to their prohibited components [20].

There is a lack of resources and poor assessment of developing trends in the usage of supplements among fitness athletes in gyms, particularly among females, as only a few studies are annually published in this regard. Considering the participation of female fitness athletes in gyms, it is essential to know about their patterns of supplement usage to develop teaching programs toward avoiding excessive and indiscriminate use of supplements [21].

There are several main questions that any athlete should ask before using supplements [3]. The reason for using them is the most crucial factor to consider. Few have the necessary teaching and skills to ask these questions [3]. It seems that coaches are the most important sources of information for athletes regarding using supplements [2, 22]. The usage rate of supplements among athletes usually varies from 40% to nearly 100% [7]. The usage of supplements by athletes in gyms is typically high, as per evidence. The overall prevalence of the use of these substances among athletes in gyms is generally above 40% [19, 23]. Differences and heterogeneity in the use of supplements among and within nations suggest that local factors (e.g., national legislation, diets, socio-economic level, and exercise culture) are important in determining the use of these substances in a gym. Consequently, the results from a specific geographic area regarding their use cannot be generalized to a broader international context. Moreover, the evidence shows different reasons for using supplements in a non-competitive context, including muscle gain, fitness, health improvement, recovery time reduction, and visual appearance improvement [19, 24, 25]. The present study aimed to investigate the prevalence of supplement consumption and its related attitudes and reasons among fitness athletes in the gyms of Kashan and its relationship with feeding behavior. Although some studies have previously explored the prevalence of supplement usage among athletes in certain cities [26–28], to the best of our knowledge, no similar studies have been conducted to explore the prevalence of the consumption of different groups of supplements and related attitudes and reasons among fitness athletes in gyms, especially in Kashan. This study takes a different approach by using a self-made questionnaire to examine the attitudes and reasons for the use of supplements and the feeding behavior of fitness athletes in the gyms of Kashan to find out if there is a relationship between supplement intake and feeding behavior among fitness athletes. The findings of this study can assist in planning future clinical research and educational programs for fitness athletes regarding the use of supplements, as well as designing appropriate diets along with the use of these substances.

## Methods

### Study design

Using a self-administered survey, this cross-sectional observational study was performed to investigate the prevalence of supplement usage and its related attitudes and reasons among fitness athletes in the gyms of all regions of Kashan. Additionally, the study sought to found the relationship between the use of these substances and the athletes’ feeding behavior. This study was conducted between May 22, 2023, and December 29, 2023.

### Participants

Participants in this study were registered through convenience sampling performed for fitness athletes in the gyms of Kashan in 2023. For enrollment, the participants had to meet inclusion criteria, including fitness athletes 15 years and older, athletes who have been gym users for at least four months continuously, and athletes who have been involved in body shaping-oriented fitness training.

According to the analyses, there are about 55 gyms in Kashan, of which 10 are for women, 2 are for men, and 43 are for both genders. The study employed a two-stage cluster sampling approach. In this way, 28 gyms were randomly selected, and then participants were randomly chosen from the selected gyms. The number of samples required in this study to estimate the proportion of fitness athletes who use supplements was calculated at 420, assuming a type I error of 0.05. The sample size was estimated according to the following formula by assuming a limited population of 6,000 fitness athletes and an accuracy of 0.03 and using the next association, in which the proportion of supplement users was taken as 0.88 according to the study of Shushtarizadeh [29];

$$d = z\_(1 - \alpha /2)\surd (p(1 - p)(N - n)/nN).$$.

### Procedures

This study was approved by the Ethics Committee of Kashan University of Medical Sciences, Kashan, Iran (IR.KAUMS.MEDNT.REC.1402.003), and all procedures were conducted following the Declaration of Helsinki. This study was performed with signed informed consent forms from all the participants. In the case of participants who were under 18 years of age, a written informed consent form was received from their parents or legal guardians. A questionnaire was given to eligible participants in the selected gyms. Athletes voluntarily completed the written questionnaire. Mentioning their names was optional for volunteers. Each questionnaire had a unique code; after completing the questionnaire, information was transferred to the software with the same unique code. Finally, data were processed using Microsoft Office Excel 2016 MSO (16.0.4266.1001) software.

### Questionnaire

Previous studies were reviewed to collect questions for the questionnaire [19, 30–34]. The questionnaire utilized in the present study was similar to the one used in previous identical studies, such as the studies conducted by Sanchez-Oliver [35, 36], Nakhaee and Pakravan [37], Darvishi [11], and Aljaloud and Ibrahim [38].

The questionnaire consisted of 48 questions in two parts. The first part included 30 questions about the athlete’s demographic records (gender, age, marital status, level of education, and income), number of training hours, participation in sports competitions, attitude toward supplements, and reasons for the consumption of supplements or lack of their consumption. In addition, participants were asked about the supplements they consume, where they purchase accessories from, their sources of information about supplements, and any side effects they may have experienced in this part. The supplements for the survey were selected based on their high consumption among athletes in similar studies [39–42], considering the region’s culture and the availability of supplements [26, 30, 31, 43].

The second part of the questionnaire analyzed the feeding behavior of athletes through 18 questions, which measured the frequency of consumption for various food groups such as beans, nuts, fruits, vegetables, dairy products, meats, oils, and snacks such as puffs and industrial fruit juices. The frequency was measured on a monthly, weekly, and daily basis.

The questionnaire used in the present study was developed for this study. It is available in Appendix A3 of the Supplementary Material File of this article.

The questionnaire developed by the researcher for the preceding items was administered to 30 participants, who completed it successfully. To ensure its reliability, the same questionnaire was given to the same group of participants after 30 days to eliminate practice and repetition effects. The participants retook the questionnaire, and the responses were collected again for comparison. Cronbach’s alpha coefficient was calculated, and external reliability was determined through the repeatability of the results. To achieve acceptable reliability, some questions were removed at each stage. The main structure of the questionnaire was then created based on the number of factors with accepted validity and reliability.

### Statistical analysis

Five experts examined the questionnaire to check its validity, and the Content Validity Ratio (CVR) and Content Validity Index (CVI) were calculated. Cronbach’s alpha and Interclass Correlation Coefficient (ICC) were used to assess the consistency of responses within a group (internal reliability) and across different raters (inter-person reliability), respectively. The recommended formula was utilized to perform a reliability study with a confidence interval of 0.95, a test power of 0.8, a minimum acceptable reliability of 0.8, and an expected reliability of 0.9. The reliability analysis of the survey items demonstrated that all measured variables were reliable, and the values of all extracted latent variables were above 0.8 (for Cronbach’s alpha).

After collecting and processing the data, the items were coded and entered into the SPSS 16 software for data analysis. The collected data were summarized using the descriptive analyses of counts, percentages, means, medians, and quartiles as continuous variables, as well as counts and rates as categorical variables. Then, the data were analyzed using Chi-square tests, taking into consideration the age and gender of the subjects. The statistical significance level was set at *p* < 0.05.

## Results

### General characteristics of the participants

The necessary information was extracted from the gyms of the regions of Kashan, and 530 questionnaires were distributed among fitness athletes. A total of 433 answered questionnaires were analyzed using the cluster random sampling method, with a response rate of 81.7%. This study included 272 male (62.8%) and 161 female (37.2%) fitness athletes. They were 15‒46 years old, and most volunteers were in the age group of 21–25 (27.2%). Data analysis revealed that the mean age of the fitness athletes was 27.6 years old.

The demographic characteristics of the participants of this study are summarized in Table 1. Most of the athletes were single (59.3%). The education level of most participants was higher than a middle school degree, and only 16.6% had a middle school degree. Table 2 presents the athletes’ general time of exercise per week and their experience of participating in competitions. In this study, 24.0% of the athletes had experience participating in competitions, 36.5% of whom had a history of participating in national and international championships. Further, most of the fitness athletes (46%) exercised for more than 6 h per week.

**Table 1:** Demographic characteristics of the participants

Demographic characteristics	Groups	Percentage (%)
Marital status	Married	40.7
	Single	59.3
Gender	Female	37.2
	Male	62.8
Education	Middle school degree	16.6
	High school degree	31.9
	Associate degree	12.7
	Bachelor’s degree	29.8
	Master’s degree	7.9
	Ph.D. degree	1.1
Income (per month)	Very low income (≤ 84 dollars)	40.6
	Low income (85–170 dollars)	31.2
	Middle income (171–250 dollars)	17.1
	High income (> 250 dollars)	11.1
Age (year)	15–20	18.0
	21–25	27.2
	26–30	19.4
	31–35	16.2
	36–40	12.0
	> 40	7.2

**Table 2:** General sports questions regarding time of exercise per week of the fitness athletes and their experience of participating in competitions

General sports questions	Groups	Percentage (%)
Total hours of exercise per week	1 h and a half	10.0
	3 h	10.9
	4–6 h	33.1
	> 6 h	46.0
Experience participating in competitions	Yes	24.0
	No	76.0
Level of competition	Regional championships	39.4
	Inter-district championships	24.0
	National championships	32.7
	International championships	3.8

### Prevalence of the use of supplements among fitness athletes

The results of the present study showed that 57.9% of fitness athletes (*n* = 251) consumed supplements.

The supplements that were examined in this research (Table 3) were categorized into four groups based on the AIS position statement according to the level of scientific evidence accessible to date [5].

**Table 3:** Classification of supplements examined in this study

Categories of supplements	Sub-categories	Examples (from the supplements considered in this study)
Group A Evidence level: Strong scientific evidence for use in specific situations in sports using evidence-based protocols	Sports foods	Whey protein, gainer, egg powder, carbohydrate powder, soybean powder, protein-rich, and energy drink
	Medical supplements	Vitamin D, Vitamin B12, vitamin B complex, multivitamin, calcium, iron, calcium + vitamin D, vitamin B9, and zinc
	Performance supplements	Caffeine, creatine, beta-alanine, aspartic acid, and theophylline
Group B Evidence level: Emerging scientific support, deserving of further research	Food polyphenols	
	Antioxidants	Vitamin C
	Tastants	
	Other	Omega-3 fatty acids, carnitine, green tea, green coffee, fish oil, and flaxseed
Group C Evidence level: Scientific evidence not supportive of benefit amongst athletes or no research undertaken to guide an informed opinion	Category A and B products used outside approved protocols	
	Named products	Vitamin E, glutamine, magnesium, amino acids, arginine, and branched-chain amino acids
	The rest	
Group D Evidence level: Banned or at high risk of contamination with substances that could lead to a positive doping test	Stimulants	Ginseng, ephedrine, amphetamine, and methylphenidate
	Prohormones and hormone boosters	Testosterone, oxymetholone, nandrolone, metandienone, insulin, and erythropoietin
	GH releasers and peptides	Growth hormone
	Beta-2 agonists	
	Selective androgen receptor modulators	
	Metabolic modulators	
	Other	

Note. GH: Growth hormone

**Fig. 1:**
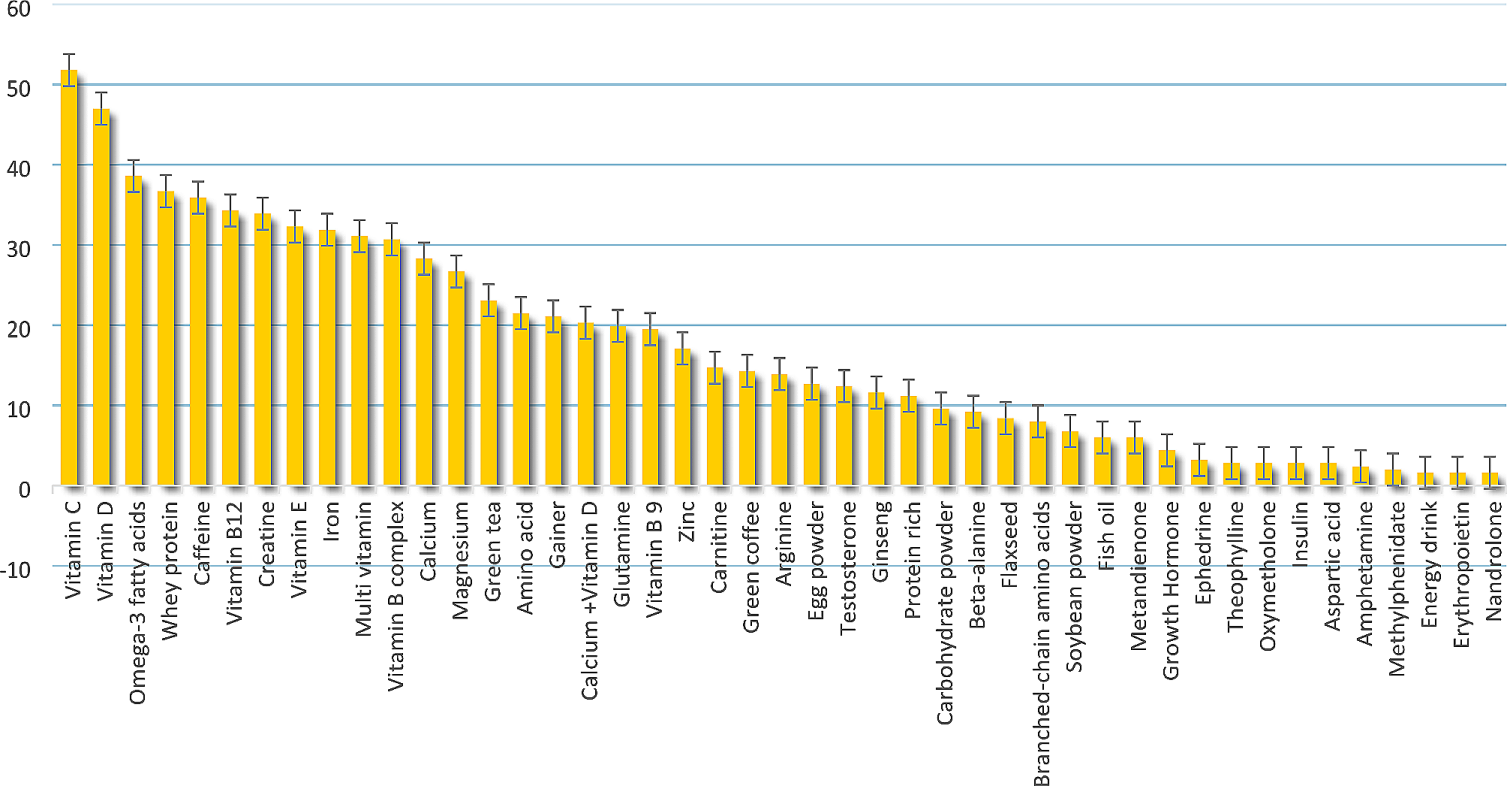
The frequency of the most commonly consumed supplements in fitness athletes

According to the results, the most frequently consumed supplements in each group are as follows (Fig. 1):

- In Group A, the most frequently consumed supplement has been vitamin D from the medical supplements sub-category (47%).
- In Group B, the most frequently consumed supplement has been vitamin C from the antioxidants sub-category (51.8%).
- In Group C, the most frequently consumed supplement has been vitamin E from the named product sub-category (32.3%).
- In Group D, the most frequently consumed supplement has been testosterone from the prohormones and hormone boosters sub-category (12.4%).

It should be noted that some of the supplements examined in this study, including testosterone, metandienone, growth hormone, oxymetholone, ephedrine, insulin, amphetamine, erythropoietin, methylphenidate, and nandrolone, are prohibited by the World Anti-Doping Agency (WADA) [44].

### Relationship between demographic variables and supplement usage

Table 4 provides the frequency of supplement consumption across different demographic variables. No significant correlation was found between the consumption of supplements and demographic factors such as age, gender, income, or marital status. The results of the study revealed that male fitness athletes tend to use supplements more frequently than females, but this difference was not significant (*p* = 0.426). In this study, 161 male fitness (59.1%) and 90 female fitness (55.9%) athletes reported consuming supplements. Among the participants, the highest percentage of supplement consumption was in the age group above 40 years old. Approximately 64.5% of the fitness athletes in this age group used various types of supplements.

**Table 4:** Relationship between demographic variables and the use of supplements

Demographic variables		Using supplements (%)	*p*-value
**Age** **(year)**	15–20	44.9	0.187
	21–25	61.0	
	26–30	63.1	
	31–35	58.6	
	36–40	55.8	
	˃ 40	64.5	
**Income** **(per month)**	Very low income (≤ 84 dollars)	58.4	0.786
	Low income (85–170 dollars)	61.4	
	Middle income (171–250 dollars)	64.1	
	High income (˃ 250 dollars)	55.8	
**Gender**	Female	55.2	0.426
	Male	59.1	
**Education**	Middle school degree	47.8	0.391
	High school degree	59.1	
	Associate degree	63.6	
	Bachelor’s degree	61.2	
	Master’s degree	50.0	
	Ph.D. degree	60.0	
**History of exercise in gyms**	< 1 year	40.0	**0.000**
	1–2 years	59.5	
	3–5 years	65.2	
	6–10 years	69.2	
	˃ 10 years	78.7	
**Marital status**	Married	54.2	0.239
	Single	60.0	
**Participating in the competitions**	Yes	69.9	**0.004**
	No	53.6	

Moreover, fitness athletes with postgraduate education levels had the highest rate of supplement consumption (63.6%). Based on the results, 60% of single and 54.2% of married participants used supplements.

The study findings demonstrated that supplement consumption was directly related to participation in competitions (*p* = 0.004) and years of exercise in gyms (*p* = 0.000). According to Tables 3 69.9% of fitness athletes, who have previously participated in competitions, used supplements. Nearly, 78.7% of fitness athletes attending gyms for over ten years consumed supplements as well. However, only 40% of the athletes attending the gym for one year or less used supplements.

### Reasons for the use of supplements

Table 5 presents data on why fitness athletes take supplements. The most prevalent reason was the desire to enhance the speed of body repair after sports activities, which was reported by 69.5% of the fitness athletes. Increasing muscle volume (68.2%) and improving body appearance (64.1%) were the second and third most common reasons for using supplements, respectively. Table 6 provides the results related to the relationship between gender and reasons for using supplements in fitness athletes. The findings of this study showed that out of eleven reasons for taking supplements that were asked in the questionnaire, eight were significantly stronger for men than for women. These reasons included compensating for nutritional deficiencies, engaging in long training, increasing muscle mass, improving accuracy and focus, reducing stress levels, enhancing body appearance, speeding up the body’s recovery after sports activities, and strengthening the immune system. On the other hand, there was no significant difference between men and women for three reasons, including increasing speed and agility, improving health, and considering others’ advice.

**Table 5:** Reasons for the use of supplements among fitness athletes

Reasons for the use of supplements	Percentage (%)
I use supplements to speed up the body’s recovery after sports activities.	69.5
I use supplements to enhance my muscle mass.	68.2
I use supplements to improve the appearance of my body.	64.1
I use supplements to prevent fatigue and to do long-term training.	63.2
I use supplements to strengthen my immune system.	63.0
I use supplements to compensate for my nutritional deficiencies.	53.2
I use supplements to improve my health.	47.3
I use supplements to increase my speed and agility.	45.0
I use supplements to improve accuracy and focus.	42.0
I use supplements to reduce my stress.	20.3
I use supplements because of others’ advice.	20.3

**Table 6:** Relationship between gender and reasons for using supplements among fitness athletes

Reasons for the use of supplements	Gender		*p*-value
	Female (%)	Male (%)	
I use supplements to compensate for my nutritional deficiencies.	42.1	64.8	**0.003**
I use supplements to prevent fatigue and to do long-term training.	54.6	75.5	**0.003**
I use supplements to increase my muscle mass.	45.3	81.8	**0.000**
I use supplements to improve accuracy and focus.	30.7	51.1	**0.007**
I use supplements to reduce my stress.	9.3	24.4	**0.013**
I use supplements to increase my speed and agility.	36.5	50.7	0.138
I use supplements to improve the appearance of my body.	49.2	73.2	**0.001**
I use supplements to speed up the body’s recovery after sports activities.	57.8	80.7	**0.001**
I use supplements to strengthen my immune system.	55.5	72.6	**0.018**
I use supplements to improve my health.	50.7	52.7	0.803
I use supplements because of others’ advice.	11.1	22.4	0.058

### Reasons for a lack of supplement usage

**Fig. 2:**
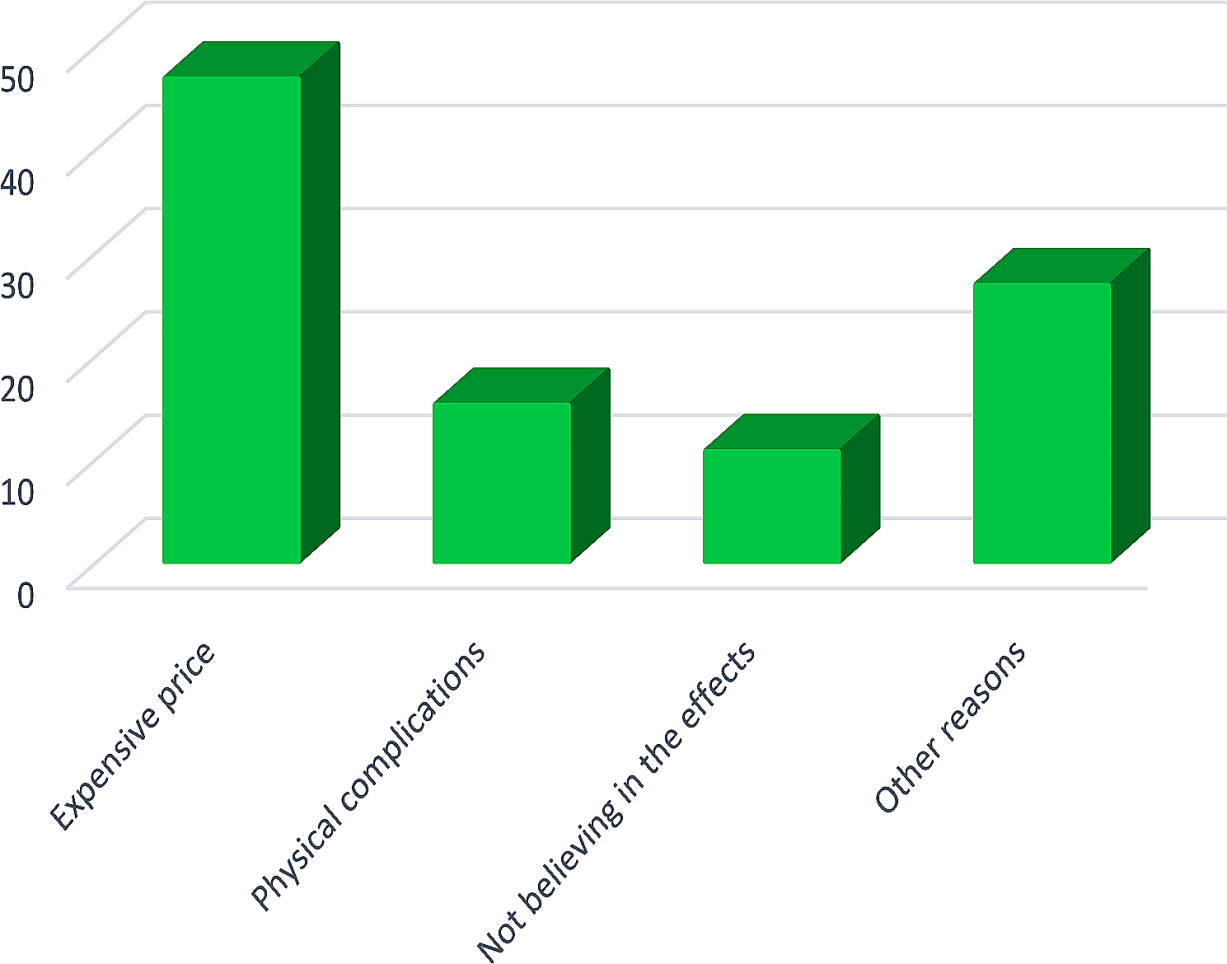
Reasons for not taking supplements among fitness athletes

In Fig. 2, the most common reasons for not taking supplements among fitness athletes are reported in percentages. The findings revealed that the most important reason for their lack of supplement consumption was the high price of supplements (46.9%), while only 15.3% avoided supplements due to side effects.

### Attitudes of fitness athletes toward supplements

Table 7 reports fitness athletes’ attitudes toward supplements. The results demonstrated that 42.8% of fitness athletes believed supplements enhanced their performance, and 21.9% thought that supplements were generally not harmful to the body. About 26.8% of fitness athletes believed exercising increases the need for supplements. On the other hand, 27% of the athletes agreed that supplements are unnecessary if they maintain a balanced diet. Furthermore, 59.8% of the athletes did not believe that supplements could replace daily food.

**Table 7:** The attitude of fitness athletes toward supplements

Attitudes	I agree (%)	I agree to some extent (%)	No idea (%)	I somewhat disagree (%)	I disagree (%)
Supplements increase the performance of athletes.	42.8	24.7	21.7	1.6	9.2
Exercise increases the human need for supplements.	26.8	25.9	27.9	4.6	14.8
The use of supplements causes a positive doping test.	7.2	12.4	48.8	6.7	24.9
Supplements generally do not harm the body.	21.9	25.6	30.0	9.7	12.8
Supplements can replace daily food.	3.8	8.5	18.2	9.7	59.8
There is no need to take supplements if you eat a balanced diet.	27.0	26.3	22.6	11.6	12.5
I would like to know more about supplements.	43.2	17.8	26.9	3.4	8.7

Almost half of the fitness athletes were interested in learning more about supplements. Additionally, only a tiny percentage of them (7.2%) believed that using supplements could cause a positive doping test.

Overall, 78.4% of fitness athletes who took supplements achieved their desired results, while 21.6% did not.

### The relationship between supplement intake and feeding behavior in fitness athletes

This study explored the connection between feeding behavior and the use of supplements among fitness athletes. The research included 18 questions, and the findings indicated that fitness athletes who take supplements tend to consume more white meat (*p* = 0.004) and nuts and seeds (*p* = 0.023) compared to athletes who do not use supplements. Instead, fitness athletes who take supplements tend to consume lower amounts of high-fat dairy products (*p* = 0.011). There was no significant difference in the consumption of various food items such as whole meal bread, white bread, raw vegetables, fruits, low-fat dairy products, beans, veal, mutton, chicken, turkey, fish, sausage, soda, fruit juice, snacks, eggs, potato, solid oil, liquid oil, tea, and coffee between fitness athletes who used supplements and who did not use them. A detailed breakdown of the consumption of each food item by athletes is available in Appendix A1 of the Supplementary Material File of this paper.

### The relationship between the use of supplements and gender in fitness athletes

**Fig. 3:**
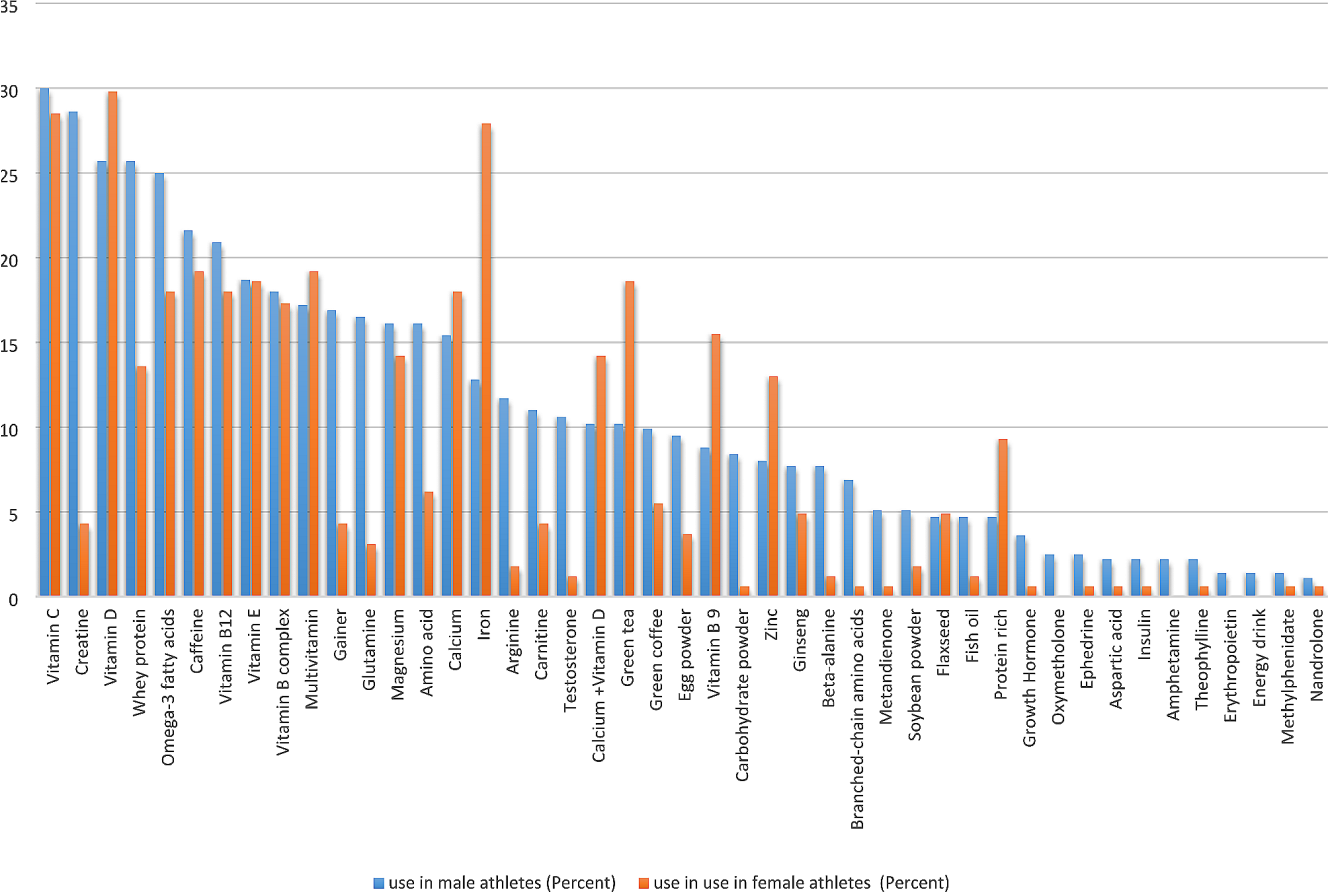
The frequency of commonly consumed supplements in male and female fitness athletes

Figure 3 shows the percentage of commonly consumed supplements in male and female fitness athletes. According to the findings, female fitness athletes used green tea (*p* = 0.003), protein-rich (*p* = 0.034), iron (*p* = 0.000), zinc (*p* = 0.044), and vitamin B9 (*p* = 0.011) significantly more than males. However, male fitness athletes used gainer (*p* = 0.000), testosterone (*p* = 0.000), metandienone (*p* = 0.016), carbohydrate powder (*p* = 0.001), beta-alanine (*p* = 0.005), creatine (*p* = 0.000), carnitine (*p* = 0.007), arginine (*p* = 0.000), glutamine (*p* = 0.000), amino acid (*p* = 0.003), branched-chain amino acids (*p* = 0.003), whey protein (*p* = 0.004), and egg powder (*p* = 0.034) significantly more than females.

No significant difference was observed between male and female fitness athletes in their use of some supplements, including aspartic acid, multivitamins, vitamin C, vitamin E, vitamin B complex, magnesium, vitamin B12, vitamin D, calcium + vitamin D, calcium, nandrolone, insulin, oxymetholone, erythropoietin, growth hormone, flaxseed, green coffee, ginseng, energy drinks, methylphenidate, amphetamines, theophylline, ephedrine, caffeine, fish oil, omega-3 fatty acids, and soybean powder. A detailed account of the usage of these supplements by male and female fitness athletes is available in Appendix A2 of the Supplementary Material File of this article.

### Places fitness athletes buy supplements

Based on the results, the pharmacy (40.4%) was the most preferred place for fitness athletes to purchase supplements, followed by supplement sales agencies (25.2%). Some fitness athletes bought supplements from city-level stores (10.1%), while others preferred online shops (9.1%), coaches (8.0%), gyms (4.7%), trusted people (1.5%), or foreign sources (1.0%).

### Important factors for fitness athletes to consider when choosing supplements

According to the present study, fitness athletes primarily choose supplements based on the brand name (40.1%) and the effectiveness of the supplements (39.2%). Other significant factors included the date of manufacture (38.3%), standard signs (37.4%), and ingredients and content (36.9%). Interestingly, the least important factor for these athletes when choosing supplements was the side effects (27.3%).

### Sources of information for fitness athletes regarding supplements

**Fig. 4:**
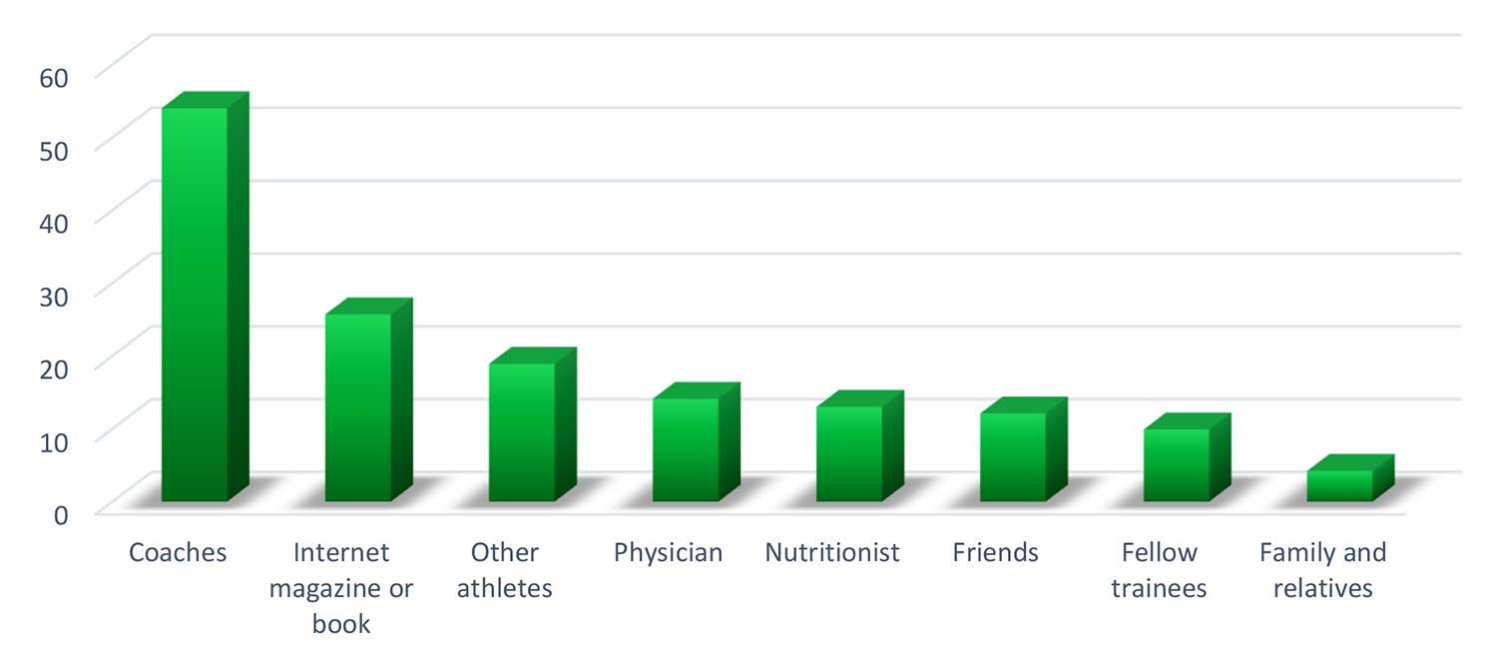
The sources of information of fitness athletes regarding supplements

Figure 4 displays the sources of information for fitness athletes regarding supplements. The most important sources of information for these athletes were coaches (54%), the internet book or magazine (25.7%), and other athletes (18.9%), respectively. Other sources of information for fitness athletes regarding supplements were physicians, nutritionists, friends, fellow trainers, family, and relatives.

### Adverse effects experienced by fitness athletes while using supplements

Based on the findings, 65.7% of those who consumed supplements reported experiencing one or more complications. These complications were hormonal disorders (16.7%), restlessness and aggression (13.1%), nausea and vomiting (11.1%), cardiovascular complications (7.1%), change in the color of urine and feces (6.3%), decreased libido (5.9%), and yellowing of the skin and eyes (5.5%), respectively.

Male fitness athletes experienced significantly more adverse effects from supplements compared to females (*p* = 0.028).

## Discussion

This study investigated the prevalence of supplement usage and related attitudes and reasons among fitness athletes in 28 gyms in Kashan in 2023 and its relationship with feeding behavior.

### Prevalence of the use of supplements among fitness athletes

The prevalence of supplement consumption among fitness athletes in this study (57.9%) is consistent with the results of other studies reporting great levels of supplement consumption in athletes [14, 22, 30, 45, 46].

Moreover, a similar prevalence was observed among athletes in the United States (71.2%) [47], Spanish athletes (64%) [48] and more than half of the Iranian athletes in previous studies [11, 29, 43] who took supplements [49].

In the present study, 161 male fitness (59.1%) and 90 female fitness (55.9%) athletes reported taking supplements. These findings are in line with those of a meta-analysis conducted by Moazi [46], indicating that the prevalence of supplements usage in Iranian athletes was 55%. Similarly, the findings of a meta-analysis performed by Kashi [43] showed that the estimated point of taking ergogenic substances in male and female athletes was 63.9 and 28.3%, respectively. Additionally, Taheri [28] found that 39.2% of the athletes used at least one supplement for strength-building purposes in sports. It is apparent that the use of supplements has increased over time.

It is notable that the prevalence of supplement usage among athletes in the present study was lower than that of some other studies among other athletes, such as endurance athletes [2, 50, 51]. For example, this rate was lower than that of the study by Graybeal [52] among endurance athletes (cyclists, runners, and triathletes), with a mean age of 39.4 years old. These differences in the results of studies could be due to the important point that the prevalence rate of supplement consumption varies between studies due to factors such as different fields of sport, varying sample sizes, age categories, gender differences, socio-economic and geographical differences, and various levels of competition among athletes [41, 50, 52, 53]. It is also notable that African and Asian athletes reported using significantly fewer supplements than athletes from other continents [54].

### Relationship between demographic variables and supplement usage

According to the findings, there was no significant correlation between the usage of supplements and the demographic variables of age, gender, income, education, and marital status. However, in the study of Taheri [28], there was a negative and significant relationship between the prevalence of stimulant drug use and age, education level, and sports history. Likewise, Azizi [40] found a significant inverse correlation between supplement consumption and education. Additionally, Jovanov [2] concluded that the use of sports supplements among young athletes from four countries increased with age. For male athletes, supplementation was the preferred choice. Similarly, Graybeal [52] demonstrated that older athletes used more supplements than younger athletes.

This variability in research findings regarding the relationship between demographic variables and the use of supplements may be attributed to local factors such as socio-economic status and exercise culture.

The findings of this study indicated that individuals who attend gyms and participate in fitness competitions for a more extended period are more likely to take supplements, which is consistent with the findings of previous studies conducted by Azizi [40] and Nakhai [37].

### The most commonly consumed supplements among fitness athletes

Vitamin C, vitamin D, omega-3 fatty acids, and whey protein were the most frequently used supplements among fitness athletes.

The most consumed supplement in Group A was vitamin D from the medical supplements sub-category, while vitamin C from the antioxidants sub-category was the most consumed supplement in Group B. As in Group C, vitamin E from the named product sub-category was the most commonly consumed supplement. In Group D, testosterone from the sub-category of prohormones and hormone boosters was the most frequently consumed supplement, which is on the list of prohibited substances by WADA [44]. These findings are consistent with those of other studies [2, 22]. Similarly, in the study by Shoshtarizadeh [29], food supplements and vitamin groups were mostly consumed among the athletes. Similarly, Darvishi et al. [11] indicated that multivitamins and vitamin C were the most popular supplements. Likewise, Rashid Lamir et al. [22] reported vitamins as the most consumed supplements.

Vitamin C was the most frequently consumed among supplement users in the present study. Scientific evidence suggests that many athletes use high-dose vitamin C supplementation to enhance athletic performance. Research on the use of vitamin C and athletic performance revealed contradictory results [55]. Some studies demonstrated positive effects associated with decreased markers of muscle damage after strong exercise with vitamin C supplementation [56]. However, some other studies have reported either neutral or negative effects of high-dose vitamin C supplementation on muscle damage, physical performance, perceived muscle soreness, and/or adaptations to exercise [57]. One recent review study has shown that long-term high-dosage supplementation with vitamin C is not recommended due to a lack of consistent data and the potential for blunted physiologic adaptations to training. The results of this study suggest that athletes should obtain their antioxidants from a nutrient-rich diet rather than relying on supplements [55]. Doses of approximately 0.2 g per day of vitamin C consumed through five or more servings of fruits and vegetables may be adequate to reduce oxidative stress without impairing training adaptations [58].

Vitamin D was the second most frequently consumed supplement among fitness athletes in the present study. Among athletes, there is a high prevalence of vitamin D deficiency, which may put them at risk of stress fractures, illness, and delayed muscle recovery. It is important to identify individuals who are vitamin D deficient and need supplementation in order to optimize performance and prevent future injury [59, 60]. Scientific evidence indicates that vitamin D deficiency can have effects on bone, muscle, respiratory, neurological, and respiratory health [61]. The results of a recent systematic review confirmed that the greatest benefit of vitamin D supplementation in elite athletes may be improving aerobic endurance, anaerobic power, and strength [62]. A treatment dosage of 50,000 IU/week for 8 weeks is recommended for patients who are vitamin D deficient or insufficient. Once the treatment regimen has been completed, the physician may choose to recheck vitamin D levels and then initiate another 8-week round of treatment if levels remain deficient [59, 63].

The third most commonly used supplement among fitness athletes in the current study was omega-3 fatty acids. In recent years, there has been increasing research attention to the role of omega-3 fatty acids in sports performance [64]. Several studies have suggested the beneficial effects of omega-3 fatty acids on performance through improved endurance capacity and delayed onset of muscle pain, as well as on markers related to improved recovery and immune modulation. Most of these studies were conducted on amateurs. The available evidence represents that omega-3 fatty acid supplementation may have beneficial effects for athletes, particularly amateurs. However, making strong deductions and clear recommendations about the use of omega-3 fatty acid supplementation is currently difficult due to the inconsistent findings of some studies [65–68].

Whey protein is another most commonly consumed supplement among fitness athletes in the current studies. The consumption of whey protein among athletes has increased globally over the past two decades [2, 32, 33, 69]. To achieve new world records, athletes require higher protein intake for improved metabolic adaptation, tissue repair, and remodeling. Balanced meal plans may not always meet the nutritional requirements of athletes. However, consuming additional protein can help fulfill their needs and is a suitable choice for those who do not have time to prepare their meals [70]. Whey protein supplement is one of the most frequent and easy-to-purchase protein supplements. Whey protein supplements are often used to recover better vital signs and physical performance in athletes. The results of a recent network meta-analysis have provided support for the effectiveness and safety of whey protein in enhancing athletes’ sports performance and aiding in recovery [71].

### Fitness athletes’ reasons for using supplements

Speeding up body repair after sports activities, building muscle volume, and improving body appearance were the most common reasons mentioned by fitness athletes for taking supplements, which is in line with the findings of previous research [28, 37]. However, in the study by Mettler et al. [72], the most frequent reasons for consuming supplements were increasing muscle mass, improving overall health, and enhancing sports performance.

Based on the results of the present study, improving body appearance was one of the main reasons for using supplements among fitness athletes, which was a greater factor than in previous studies [2, 37]. This result may be due to the increased societal pressure and media attention toward achieving a perfect body shape, especially for athletes in recent years. It may be a reason for increasing the desire of athletes to use supplements that enhance physical appearance, especially steroids.

According to a study conducted by Finamore et al. [73], male athletes use supplements to enhance muscle volume and strength, improve muscle recovery, boost performance, reduce fat mass, and alleviate fatigue after exercise. It appears that women had the only meaningful motivations for using supplements, which were primarily for medical purposes. In the same way, in the present study, all the motives for taking supplements were significantly higher in men than in women, and only three reasons did not have a significant difference between male and female fitness athletes. They included improving health, following the advice of others, and increasing speed and agility.

### Fitness athletes’ reasons for not using supplements

The findings showed that the most important reason for the lack of supplement usage among fitness athletes was their expensive price (46.9%). Only a tiny percentage of fitness athletes (15.3%) avoided supplements due to physical complications. These results contradict those of the study by Jovanov et al. [2], in which 41.8% of supplement non-user athletes stated that they did not need them, and 21% believed that supplements were unhealthy [2].

This heterogeneity in the findings of studies regarding the athletes’ reasons for not using supplements may be attributed to local factors such as socio-economic status, exercise culture, and consciousness about supplements.

### Attitudes of fitness athletes toward supplements

In the present study, 42.8% of the fitness athletes totally agreed with the attitude that supplements improve their performance, which conforms to the results of Nakhaee and Pakravan [37], indicating the most important attitude of athletes regarding the use of supplements. Athletes in the current study and the above-mentioned study had the attitude that exercise increases the human need for supplements. However, Aljaloud and Ibrahim [38] showed that most athletes believed that supplements were beneficial for their health and enhanced their endurance.

It seems that various factors can influence the attitude of athletes toward supplements, including awareness about supplements and the individual experiences of athletes, demonstrating heterogeneity in the findings of different studies in this regard.

### The relationship between supplement intake and feeding behavior in fitness athletes

In this study, a correlation was found between feeding behavior and the use of supplements in fitness athletes. The findings revealed that fitness athletes who consume supplements follow a healthier diet with more white meat, seeds, and nuts and fewer high-fat dairy products compared to those who do not use supplements. Likewise, Bianco et al. [74] reported that athletes who take supplements consume significantly more protein-rich foods, such as fish, eggs, beans, milk, yogurt, and especially meat. Nonetheless, athletes who did not take supplements mostly consumed snacks and sweets.

It seems that athletes who use supplements are primarily motivated by the desire to enhance their performance, increase the speed of body repair after exercise, improve their physical appearance, boost their immune system, and maintain good health [28, 37]. As a result, athletes who take supplements usually prioritize maintaining a healthy and balanced diet because they value their performance, physical appearance, and health.

### The relationship between gender and the use of supplements in fitness athletes

Based on the results, there was a significant correlation between gender and the use of supplements in fitness athletes. The findings revealed that the types of consumed supplements significantly differed between male and female fitness athletes. Specifically, men consumed more protein and steroids than their female counterparts. In contrast, women used more supplements such as vitamin B9, zinc, iron, and green tea. This aligns with the findings of Aguilar-Navarro et al. [53] and Mettler et al. [72]. Similarly, in the study by Finamore et al. [73], male athletes used more supplements than women. Men were more likely to consume amino acid supplements, creatine, branched-chain amino acids, protein powders, carnitine, and energy drinks than women.

### Places where fitness athletes buy supplements

According to the findings of this study, fitness athletes considered pharmacies, supplement sales agencies, and city-level stores as the most important places to purchase supplements. This aspect has not been thoroughly examined in previous studies [22]. However, it is crucial to consider the purchase location of supplements, as it can impact the quality of the product, the attitude of athletes, and their usage patterns. Therefore, it is important to take this factor into account in future research and education plans.

### Important items for fitness athletes to choose a supplement

The research findings revealed that the brand name is the most crucial factor that fitness athletes consider when selecting supplements, while the potential side effects of them are the least important, which is consistent with the results of Kargarfard [75] and Taheri et al. [28], indicating that athletes lack sufficient knowledge to pick an appropriate supplement.

### Sources of information for fitness athletes regarding supplements

The most important sources of information for fitness athletes regarding the use of supplements were coaches, books, internet magazines, other athletes, physicians, nutritionists, friends, training partners, family, and relatives, respectively. Further, in the studies by Jovanov et al. [2], Finamore et al. [73], and Bianco et al. [74], coaches were identified as the most important source of information for athletes regarding supplements. In addition, similar results were found in the meta-analysis study conducted by Halabchi et al. [31], highlighting the necessity of providing coaches with training about supplements and advising them to consult a nutritionist to choose the best supplements for each athlete.

### Adverse effects of supplements on fitness athletes

The results of this study indicated that many fitness athletes who took supplements suffered from at least one complication. These complications included hormonal disorders, restlessness, aggression, nausea and vomiting, cardiovascular complications, changes in urine and feces color, decreased libido, and yellowing of the skin and eyes. Additionally, the findings showed that male fitness athletes experienced more adverse effects from supplements than their female counterparts.

Similarly, in the study by Bijeh et al. [26], most athletes who took anabolic steroids experienced side effects, including acne, water retention, testicular atrophy, and hair loss.

Unfortunately, many studies on supplement usage did not examine the rate of adverse effects experienced by athletes after using them [38, 40, 53].

### Limitations and strengths of the present study

There were some strong points in our study. To date, the present study, to the best of our knowledge, is the first to evaluate the prevalence of supplement usage and related attitudes and reasons and its relationship with feeding behavior among fitness athletes in the gyms of Kashan. The data from the present study were analyzed using Chi-square tests, taking into consideration the age and gender of the subjects. This study analyzed a greater number of supplements compared to similar studies. In addition, this study investigated important items for fitness athletes to choose supplements, sources of information for athletes about them, places to buy them, and adverse effects experienced after using supplements. However, the present study has some limitations. Firstly, we could only screen some of the gyms in Kashan. Secondly, the overall response rate of 81.7% did not exclude a certain selection bias. Nevertheless, we managed to include more subjects than many other studies [7], and the available indicator (average age) does not speak against the hypothesis that the study population might more or less represent the population of the screened gyms.

### Suggestions for future studies

The fitness athletes in this study experienced many side effects, and there was also evidence of hidden contamination in supplements. As a result, it is suggested that future researchers pay more attention to the issue of the side effects of using supplements for fitness athletes. Furthermore, considering that various supplements have different physical and psychological side effects, it is necessary for the researchers to design a suitable questionnaire for a more detailed examination of the side effects experienced by supplement users.

It is recommended that more surveys be conducted on the coaches’ knowledge and consciousness of supplements in the future. Particularly, these surveys should focus on the coaches’ knowledge of the potential side effects of supplements. This is especially important because a considerable number of fitness athletes rely on their coaches for information about the use of supplements.

## Conclusion

Our findings showed that the use of supplements is common among fitness athletes in the gyms in Kashan. Among the supplement users, vitamin C, vitamin D, omega-3 fatty acids, and whey protein were consumed most frequently. An increase in the speed of body repair after exercise was the main reason for using supplements. Fitness athletes mostly relied on their coaches for information regarding supplements. Furthermore, almost half of the fitness athletes completely agreed with the attitude that the use of supplements improves athletes’ performance.

In this study, a relationship was observed between feeding behavior and the use of supplements in fitness athletes. Based on the results, fitness athletes who took supplements consumed more white meat, seeds, and nuts and fewer high-fat dairy products than athletes who did not take them. These findings indicate the necessity of informing fitness athletes about using supplements. The findings of this study can provide valuable insights for planning future clinical studies and educational programs on supplements for fitness athletes.

### Electronic supplementary material

Below is the link to the electronic supplementary material.

Supplementary Material 1

## Data Availability

The datasets generated and/or analyzed during the current study are not publicly available due to the rules and regulations of the Research Center for Biochemistry and Nutrition in Metabolic Diseases at the Kashan University of Medical Sciences, but are available from the corresponding author upon reasonable request.

## Abbreviations

AIS: Australian Institute of Sports
WADA: World Anti-Doping Agency
