# Supplementary material for: Prevalence of supplement usage and related attitudes and reasons among fitness athletes in the gyms of Kashan and its relationship with feeding behavior: a cross-sectional study
Source: BMC Sports Sci Med Rehabil. 2024 Jul 8;16:150. doi: 10.1186/s13102-024-00940-3 (PMC11229390; doi:10.1186/s13102-024-00940-3)
Supplement: Supplementary file 1 — Supplementary Material 1 [file 13102_2024_940_MOESM1_ESM.docx]

**Supplementary Material:**

**Appendix A1**. Full description of the consumption of foodstuffs in athletes

| Foodstuffs | | Supplements use | | p-value |
| --- | --- | --- | --- | --- |
|  |  | Yes (%) | No (%) |  |
| Whole meal bread | never | 7.4 | 9.4 | 0.617 |
|  | 1-3 times a month | 11.9 | 15.2 |  |
|  | 1-2 times a week | 26.3 | 21.1 |  |
|  | 3-4 times a week | 25.9 | 24.1 |  |
|  | everyday | 28.3 | 30 |  |
| White bread | never | 17.2 | 13.5 | 0.100 |
|  | 1-3 times a month | 23.3 | 25.2 |  |
|  | 1-2 times a week | 31.9 | 23.5 |  |
|  | 3-4 times a week | 14.3 | 16.4 |  |
|  | everyday | 13.1 | 21.1 |  |
| Raw vegetables | never | 5.7 | 9.3 | 0.453 |
|  | 1-3 times a month | 15.9 | 14.5 |  |
|  | 1-2 times a week | 26.1 | 26.1 |  |
|  | 3-4 times a week | 27.7 | 22.0 |  |
|  | everyday | 24.4 | 27.9 |  |
| Fruit | never | 1.6 | 1.7 | 0.436 |
|  | 1-3 times a month | 7.4 | 4.0 |  |
|  | 1-2 times a week | 10.3 | 14.3 |  |
|  | 3-4 times a week | 25.6 | 28.1 |  |
|  | everyday | 54.9 | 51.7 |  |
| High-fat dairy products | never | 20.1 | 12.6 | **0.011** |
|  | 1-3 times a month | 24.2 | 29.3 |  |
|  | 1-2 times a week | 30.8 | 23.5 |  |
|  | 3-4 times a week | 9.8 | 19.5 |  |
|  | everyday | 14.8 | 14.9 |  |
| Low-fat dairy products | never | 53.2 | 3.4 | 0.805 |
|  | 1-3 times a month | 13.9 | 12.7 |  |
|  | 1-2 times a week | 22.5 | 20.3 |  |
|  | 3-4 times a week | 30.3 | 34.3 |  |
|  | everyday | 27.8 | 29.0 |  |
| Beans | never | 4.9 | 2.3 | 0.559 |
|  | 1-3 times a month | 14.4 | 17.3 |  |
|  | 1-2 times a week | 35.3 | 31.7 |  |
|  | 3-4 times a week | 30.0 | 32.3 |  |
|  | everyday | 15.2 | 16.1 |  |
| Veal and mutton | never | 5.3 | 4.6 | 0.414 |
|  | 1-3 times a month | 16.8 | 15.0 |  |
|  | 1-2 times a week | 37.7 | 32.3 |  |
|  | 3-4 times a week | 31.1 | 33.5 |  |
|  | everyday | 9.0 | 14.4 |  |
| Chicken, turkey, and fish | never | 1.6 | 5.7 | **0.004** |
|  | 1-3 times a month | 18.9 | 15.5 |  |
|  | 1-2 times a week | 30.8 | 40.8 |  |
|  | 3-4 times a week | 30.4 | 29.3 |  |
|  | everyday | 18.1 | 8.6 |  |
| Sausage | never | 42.6 | 34.6 | 0.075 |
|  | 1-3 times a month | 31.5 | 34.1 |  |
|  | 1-2 times a week | 18.8 | 23.6 |  |
|  | 3-4 times a week | 3.6 | 6.9 |  |
|  | everyday | 3.2 | 0.5 |  |
| Nuts and seeds | never | 2.0 | 6.3 | **0.023** |
|  | 1-3 times a month | 21.3 | 22.4 |  |
|  | 1-2 times a week | 26.6 | 31.6 |  |
|  | 3-4 times a week | 25.8 | 25.8 |  |
|  | everyday | 24.1 | 13.7 |  |
| Soda and fruit juice | never | 39.1 | 30.6 | 0.181 |
|  | 1-3 times a month | 26.5 | 23.2 |  |
|  | 1-2 times a week | 22.8 | 29.5 |  |
|  | 3-4 times a week | 8.1 | 11.3 |  |
|  | everyday | 3.2 | 5.1 |  |
| Snacks | never | 22.7 | 21.1 | 0.876 |
|  | 1-3 times a month | 34.5 | 30.8 |  |
|  | 1-2 times a week | 26.8 | 30.2 |  |
|  | 3-4 times a week | 10.9 | 12.5 |  |
|  | everyday | 48.7 | 5.1 |  |
| Egg | never | 2.0 | 1.7 | 0.183 |
|  | 1-3 times a month | 8.1 | 7.5 |  |
|  | 1-2 times a week | 18.2 | 27.1 |  |
|  | 3-4 times a week | 36.1 | 36.9 |  |
|  | everyday | 35.3 | 26.5 |  |
| Potato | never | 4.8 | 2.8 | 0.501 |
|  | 1-3 times a month | 17.1 | 15.0 |  |
|  | 1-2 times a week | 31.0 | 38.1 |  |
|  | 3-4 times a week | 29.7 | 26.0 |  |
|  | everyday | 17.1 | 17.9 |  |
| Solid oil | never | 32.5 | 28.7 | 0.131 |
|  | 1-3 times a month | 22.6 | 23.5 |  |
|  | 1-2 times a week | 29.6 | 23.5 |  |
|  | 3-4 times a week | 7.4 | 14.3 |  |
|  | everyday | 7.8 | 9.7 |  |
| Liquid oil | never | 16.8 | 22.4 | 0.544 |
|  | 1-3 times a month | 16.8 | 14.9 |  |
|  | 1-2 times a week | 23.3 | 22.4 |  |
|  | 3-4 times a week | 21.3 | 22.9 |  |
|  | everyday | 21.7 | 17.2 |  |
| Tea and coffee | never | 2.4 | 4.5 | 0.453 |
|  | 1-3 times a month | 9.3 | 6.2 |  |
|  | 1-2 times a week | 7.3 | 10.2 |  |
|  | 3-4 times a week | 16.6 | 16.4 |  |
|  | everyday | 64.2 | 62.5 |  |

**Appendix A2**. Full description of the use of supplements in female and male athletes

| Supplements | use in male athletes (%) | use in female athletes (%) | P-value |
| --- | --- | --- | --- |
| Vitamin C | 30.0 | 28.5 | 0.980 |
| Creatine | 28.6 | 4.3 | **0.000** |
| Vitamin D | 25.7 | 29.8 | 0.103 |
| Whey protein | 25.7 | 13.6 | **0.004** |
| Omega-3 fatty acids | 25.0 | 18.0 | 0.144 |
| Caffeine | 21.6 | 19.2 | 0.802 |
| Vitamin B12 | 20.9 | 18.0 | 0.678 |
| Vitamin E | 18.7 | 18.6 | 0.718 |
| Vitamin B complex | 18.0 | 17.3 | 0.842 |
| Multivitamin | 17.2 | 19.2 | 0.341 |
| Gainer | 16.9 | 4.3 | **0.000** |
| Glutamine | 16.5 | 3.1 | **0.000** |
| Magnesium | 16.1 | 14.2 | 0.821 |
| Amino acid | 16.1 | 6.2 | **0.003** |
| Calcium | 15.4 | 18.0 | 0.263 |
| Iron | 12.8 | 27.9 | **0.000** |
| Arginine | 11.7 | 1.8 | **0.000** |
| Carnitine | 11.0 | 4.3 | **0.007** |
| Testosterone | 10.6 | 1.2 | **0.000** |
| Calcium +Vitamin D | 10.2 | 14.2 | 0.107 |
| Green tea | 10.2 | 18.6 | **0.003** |
| Green coffee | 9.9 | 5.5 | 0.156 |
| Egg powder | 9.5 | 3.7 | **0.034** |
| Vitamin B9 | 8.8 | 15.5 | **0.011** |
| Carbohydrate powder | 8.4 | 0.6 | **0.001** |
| Zinc | 8.0 | 13.0 | **0.044** |
| Ginseng | 7.7 | 4.9 | 0.346 |
| Beta-alanine | 7.7 | 1.2 | **0.005** |
| Branched-chain amino acids | 6.9 | 0.6 | **0.003** |
| Metandienone | 5.1 | 0.6 | **0.016** |
| Soybean powder | 5.1 | 1.8 | 0.112 |
| Flaxseed | 4.7 | 4.9 | 0.792 |
| Fish oil | 4.7 | 1.2 | 0.065 |
| Protein rich | 4.7 | 9.3 | **0.034** |
| Growth hormone | 3.6 | 0.6 | 0.103 |
| Oxymetholone | 2.5 | 0.0 | 0.053 |
| Ephedrine | 2.5 | 0.6 | 0.266 |
| Aspartic acid | 2.2 | 0.6 | 0.427 |
| Insulin | 2.2 | 0.6 | 0.427 |
| Amphetamine | 2.2 | 0.0 | 0.092 |
| Theophylline | 2.2 | 0.6 | 0.427 |
| Erythropoietin | 1.4 | 0.0 | 0.300 |
| Energy drink | 1.4 | 0.0 | 0.300 |
| Methylphenidate | 1.4 | 0.6 | 0.659 |
| Nandrolone | 1.1 | 0.6 | 1.000 |

**Appendix A3.** Full version of the questionnaire used in the present study

1- If you wish, please write your name and surname: ............

2- Please write the name of the sports field you do in the gym: ........

3- Please write your age (year): …….

4- Please specify your gender: □Female □Male

5- Please specify your marital status: □Married □Single

6- Please specify your age (year):

□20-15 □21-25 □26-30 □31-35 □36-40 □˃40

7- Please specify your education level:

□ Middle school degree □ High school degree □Associate degree □Bachelor's degree □Master's degree □ Ph.D. degree

8- How much is your monthly income?

□ Very low income (≤ 84 dollars) □ Low income (85-170 dollars)

□ Middle income (171-250 dollars) □ High income ( ˃ 250 dollars)

9- How long have you been working out in gyms?

□Less than a year □1-2 years □3-5 years □6-10 years □Above 10 years

10- How many hours do you usually exercise per week in the gym?

□1 hour and a half □3 hours □4-6 hours □˃ 6 hours

11- Have you ever participated in competitions? □Yes □No

12- If the answer is yes, in which of the levels of the following competitions have you participated so far?

□ Regional championships □ Inter-district championships □ National championships □ International championships

13- If you use supplements, please answer the following questions.

A- I use supplements to compensate for my nutritional deficiencies. ​

□Yes □No

B- I use supplements to prevent fatigue and to do long training. ​​​​​

□Yes □No

C- I use supplements to increase my muscle mass. ​​​​​

□Yes □No

D- I use supplements to improve accuracy and focus. ​​​​​

□Yes □No

E- I use supplements to reduce my stress. ​​​​​

□Yes □No

F- I use supplements to increase my speed and agility. ​​​​​

□Yes □No

G- I use supplements to improve the appearance of my body. ​​​​​

□Yes □No

H- I use supplements to speed up the body's recovery after sports activities. ​​​​​

□Yes □No

I- I use supplements to strengthen my immune system. ​​​​​

□Yes □No

J- I use supplements to improve my health.

□Yes □No

K- I use supplements because of others' advice.

□Yes □No

14- Supplementsincrease the performance of athletes.

□I agree □I agree to some extent □No idea □I somewhat disagree □I disagree

15- Exercise increases the human need for supplements.

□I agree □I agree to some extent □No idea □I somewhat disagree □I disagree

16- The use of supplements causes a positive doping test.

□I agree □I agree to some extent □No idea □I somewhat disagree □I disagree

17- Supplements generally do not harm the body.

□I agree □I agree to some extent □No idea □I somewhat disagree □I disagree

18- Supplements can replace daily food.

□I agree □I agree to some extent □No idea □I somewhat disagree □I disagree

19- In case of consuming a balanced diet, it is not necessary to consume supplements.

□I agree □I agree to some extent □No idea □I somewhat disagree □I disagree

20- I would like to know more about supplements.

□I agree □I agree to some extent □No idea □I somewhat disagree □I disagree

21- If you do not use supplements, please specify the most important reason for not using them.

□high price □ physical complications □ not believing in the effects □ Other

22- Which of the following places do you buy supplements from?

□ online shops □pharmacy □ city-level stores □ sales agencies

23- If you get supplements from another place, please write it: .................

24- So far, you have obtained more information about supplements from which of the following?

□ physicians □ nutritionists □ family and relatives □ other athletes

□ coaches □ internet book or magazine □ friends □ fellow trainers

25- Which of the following is important for you to choose a supplement? (you can select more than one option)

□ standard signs □ brand name □ ingredients and content □ date of manufacture

□ side effects □ effectiveness

26- Which of the following side effects have you experienced by taking supplements? (you can select more than one option)

□ restlessness and aggression □ change in color of urine and feces □ decreased libido

□ cardiovascular complications □ nausea and vomiting □ yellowing of the skin and eyes □ hormonal disorders

27- Did you get the desired results after taking supplements?

□Yes □No

28- Please put a mark on that house from each of the supplements in the table below that you use.

| Caffeine | Green tea | Green coffee | Creatine | Whey protein | Egg powder |
| --- | --- | --- | --- | --- | --- |
| Omega-3 fatty acids | Fish oil | Glutamine | Gainer | Carbohydrate powder | Ginseng |
| Vitamin B9 | Vitamin D | Vitamin E | Vitamin B12 | Vitamin B complex | Vitamin C |
| Zinc | Calcium | Iron | Magnesium | Calcium +Vitamin D | Multivitamin |
| Arginine | Amino acid | Beta-alanine | Theophylline | Amphetamine | Aspartic acid |
| Growth hormone | Testosterone | Erythropoietin | Carnitine | Branched-chain amino acids | Protein rich |
| Ephedrine | Metandienone | Oxymetholone | Nandrolone | Methylphenidate | Insulin |
|  | | | Energy drink | Soybean powder | Flaxseed |

29- If you use other supplements besides the above items, please write down their names: .................

30- If you use gainer, please write its name: …….

31-How many times a month or week do you use whole grain breads?

□never □1-3 times a month □1-2 times a week □ 3-4 times a week □ every day

32- How many times a month or week do you use white breads?

□never □1-3 times a month □1-2 times a week □ 3-4 times a week □ every day

33- How many times a month or week do you use raw vegetables?

□never □1-3 times a month □1-2 times a week □ 3-4 times a week □ every day

34- How many times a month or week do you use fruit?

□never □1-3 times a month □1-2 times a week □ 3-4 times a week □ every day

35- How many times a week or month do you use beans?

□never □1-3 times a month □1-2 times a week □ 3-4 times a week □ every day

36- How many times in a month or week do you use high-fat dairy products?

□never □1-3 times a month □1-2 times a week □ 3-4 times a week □ every day

37- How many times in a month or week do you use low-fat dairy products?

□never □1-3 times a month □1-2 times a week □ 3-4 times a week □ every day

38-How many times a week or a day do you use chicken, fish and turkey?

□never □1-3 times a month □1-2 times a week □ 3-4 times a week □ every day

39- How many times a week or month do you use veal and mutton?

□never □1-3 times a month □1-2 times a week □ 3-4 times a week □ every day

40-How many times a week or month do you use sausages?

□never □1-3 times a month □1-2 times a week □ 3-4 times a week □ every day

41- How many times a week or month do you use nuts and seeds including all kinds of seeds, almonds, peanuts, walnuts, pistachios, hazelnuts?

□never □1-3 times a month □1-2 times a week □ 3-4 times a week □ every day

42-How many times a week do you use soda and fruit juice?

□never □1-3 times a month □1-2 times a week □ 3-4 times a week □ every day

43- How many times a week or day do you use snacks including all kinds of cakes, sweets, biscuits, chocolate, chips and puffs?

□never □1-3 times a month □1-2 times a week □ 3-4 times a week □ every day

44- How many times a week or month do you use egg?

□never □1-3 times a month □1-2 times a week □ 3-4 times a week □ every day

45- How many times a week or month do you use potato?

□never □1-3 times a month □1-2 times a week □ 3-4 times a week □ every day

46- How many times a week or month do you use solid oils?

□never □1-3 times a month □1-2 times a week □ 3-4 times a week □ every day

47-How many times a week or month do you use liquid oils?

□never □1-3 times a month □1-2 times a week □ 3-4 times a week □ every day

48-How many times a week or month do you use tea and coffee?

□never □1-3 times a month □1-2 times a week □ 3-4 times a week □ every day
